# Maternal Factors Associated with Non-Exclusive Breastfeeding in Haitian Immigrant Women in Southern Chile

**DOI:** 10.3390/nu14153173

**Published:** 2022-08-02

**Authors:** Alejandra Rodríguez-Fernández, Ximena Sanhueza-Riquelme, Gloria Cárcamo-Vargas, Julio Parra-Flores, Ana Lizette Rojas-Rodríguez, Marcela Ruíz-De la Fuente, Eduard Maury-Sintjago

**Affiliations:** 1 Department of Nutrition and Public Health, Universidad del Bío-Bío, Chillan 3780000, Chile; alrodriguez@ubiobio.cl (A.R.-F.); xsanhue@ubiobio.cl (X.S.-R.); gcarcamo@ubiobio.cl (G.C.-V.); juparra@ubiobio.cl (J.P.-F.); 2 GABO—Grupo de investigación en Auxología, Crecimiento y Desarrollo, Universidad del Bío-Bío, Chillan 378000, Chile; 3 Carrera de Medicina, Facultad de Ciencias de la Salud, Universidad Técnica Particular de Loja, Loja 110102, Ecuador; alrojas4@utpl.edu.ec

**Keywords:** maternal factors, breastfeeding, immigrants, Haitian

## Abstract

There is limited knowledge concerning factors that affect non-exclusive breastfeeding (NEBF) practices in immigrant populations, especially in Latin America. The objective of the present study was to determine the association between maternal factors and the prevalence of NEBF in Haitian immigrant women in southern Chile. This was an analytical cross-sectional study. The probabilistic sample consisted of 173 Haitian women who gave informed consent. Sociodemographic and dietary-nutritional information was collected from all participants. Bivariate (χ^2^) and multivariate (logistic regression) inferential statistics were applied. All analyses were performed with the STATA 16.0 statistical software, and the significance level was established as α < 0.05. The prevalence of EBF at 6 mo was 54.3%. Maternal factors associated with a lower prevalence of EBF were not having permanent residency (OR: 2.34, CI: 2.18–2.83), residency <12 months (OR: 2.23, CI: 2.09–2.78), limited knowledge of breastfeeding (OR: 1.96; CI: 1.81–2.27), and low educational attainment (OR: 1.78; CI: 1.61–2.11). The protective factors were employment (OR: 0.36, CI: 0.28–0.40), access to basic services (OR: 0.32; CI: 0.22–0.48), and Spanish proficiency (OR: 0.29; CI: 0.20–0.51). Haitian immigrant women without legal residency, recently arrived, with low educational attainment, and poor knowledge of breastfeeding have more risk of not providing exclusive breastfeeding. Targeted interventions for mothers with these risk factors may help improve EBF rates.

## 1. Introduction

Migration is a global phenomenon that mobilizes more than 270 million people (3.5% of the world population) across all continents [1]. Immigration in Chile has increased by 400% since 2005, and Haitian immigrants are one of the main groups [2]. The migration process in itself has been considered an important determinant of health that affects each phase of the process (preparation, mobilization, settlement, and return); it is also aggravated by such factors as poverty, sex, and migratory status [3,4]. Given this scenario, it is necessary to conduct research on the general health, dietary-nutritional status, and child-rearing patterns of migrant groups throughout the life cycle, especially during childhood.

Breast milk is the most complete food of all; it is rich in essential nutrients and other bioactive components, which are perfectly adapted to the baby and are beneficial for the mother-child dyad [5]. In addition, breast milk promotes adequate nutrition during infancy and early childhood by ensuring that children reach their full potential for growth, health protection, and development [6].

According to the World Health Organization, breastfeeding (BF) should be exclusive until 6 months old and subsequently complemented with other foods until approximately 2 years of age [7]. There is evidence that exclusive breastfeeding (EBF) decreases the risk of necrotizing enterocolitis, diarrhea, respiratory tract infections, otitis media, asthma, malocclusion, overnutrition, and mortality, as well as higher performance on intelligence tests [8,9].

The prevalence of EBF varies widely among countries; high-income countries have a prevalence of <20%, while it is ≥45% in middle- and low-income countries. The prevalence of EBF in Chilean and Haitian women is 55% and 41%, respectively [10,11]. Findings on EBF prevalence in immigrant women are still scarce and contradictory; however, it has been reported that immigrant women have a higher prevalence of initiating BF than host country nationals [12,13]. A recent meta-analysis that included 29 studies (1,539,659 women in 14 countries) found no association between immigrant status and initiation and duration of EBF [14]. Other authors have described that psychosocial, biological, and cultural aspects are the main causes of terminating EBF [15,16,17,18].

There are no reports in Chile on the prevalence of NEBF in Haitian immigrant women or conditioning factors. Therefore, the objective of the present study was to determine the association between maternal factors and the prevalence of EBF in Haitian immigrant women in southern Chile.

## 2. Materials and Methods

This was an analytical cross-sectional study. The probabilistic sample (95% confidence interval and 3% precision) consisted of 173 Haitian mothers living in southern Chile (Ñuble, Bío-Bío, and Maule Regions). The subjects were selected from a list of community (health, education, social service) and religious centers, which were identified by key informants. From this database, a code was assigned to each participant, and subsequently, using statistical software, a random selection was made. When a selected subject was unable to participate, he was replaced by another randomly selected subject. To complete the sample size, 196 women were contacted. The study was reviewed and approved by the Bioethics and Biosecurity Committee of the Universidad del Bío-Bío. All participants provided informed consent and their anonymity was protected.

Data were collected through a sociodemographic survey that included the following items: exclusive breastfeeding (EBF) until 6 months old (yes/no), maternal age (<35 years, ≥35 years), marital status (married or common-law partner, single), educational attainment (≤12 years, >12 years), parity (number of live births (1, 2, ≥3), duration of residency (≤12 months, >12 months), immigrant status (undocumented, permanent residency), employed (yes/no), Spanish proficiency (yes/no), and access to basic services (yes/no). At the time of data collection, all mothers had children >6 months old. The survey was in “*Kreyòl ayisyen*” (translated by a certified Haitian native speaker) to facilitate its application.

Weight status (WS) was determined by a certified nutritionist using anthropometric measurements that were evaluated with a TANITA scale (weight) and SECA stadiometer (height). The WS was classified according to body mass index (BMI, kg/m^2^) and the WHO reference cut-off points for BMI (<18.5 underweight, 18.5–24.9 normal, and ≥25.0 overnutrition, which includes overweight and obesity) [19].

Knowledge of exclusive breastfeeding (EBF) and complementary feeding was determined by designing a test to evaluate the knowledge of Haitian mothers. The survey consisted of seven items: (1) benefits of breastfeeding (BF), (2) EBF, (3) BF position, (4) alternatives to EBF, (5) age at which complementary feeding is introduced, (6) milk formulas provided by the Ministry of Health, and (7) commercial milk formulas [20]. The scoring range was 0–14; an intermediate-high level was ≥8.0 score. The test was validated by a panel of experts (two nutritionists and one biologist with expertise in biostatistics); the internal consistency was 0.94 (Cronbach’s alpha).

Descriptive and inferential statistics were applied. Measures of central tendency, dispersion, percentage, and frequency were used for the description according to the type of variables. The bivariate inference included the χ ^2^ tests. A multivariate logistic binary regression model adjusted by age was used to identify factors associated with NEBF. The significance level was α < 0.05. All analyses were performed with STATA 16.0.

## 3. Results

The sociodemographic characteristics of the sample showed that most participants were <35 years old, single, with >12 years education, 1 child, and >12 months residency in Chile. Some 79.2% had permanent residency and most were employed and had access to basic services. The majority showed limited Spanish proficiency and a WS of overnutrition. Regarding BF, the prevalence of EBF was 54.3%, and 72.8% of the participants had adequate knowledge of nutrition (see Table 1).

Table 2 shows that the highest EBF values were related to higher educational attainment (84%, *p* = 0.029), longer residency in Chile (88%, *p* = 0.018), resident immigrant status (86%, *p* = 0.008), employed (82%), limited Spanish proficiency (73%, *p* = 0.025), and adequate knowledge of BF (80%, *p* = 0.027).

Table 3 shows the results of the logistic regression model, which indicates that the probability of non-exclusive breastfeeding (NEBF) increases almost twofold when educational attainment is lower (OR: 1.78; IC: 1.61–2.11). Likewise, residency < 12 months increased the risk of NEBF by 2.2 times compared with those with longer residency. A similar situation occurred with immigrant status because being undocumented increased the likelihood of NEBF by more than twofold (OR: 2.23; IC: 2.09–2.78). The lack of knowledge of BF increased the risk of NEBF up to twofold (OR: 1.96; IC: 1.81–2.27). The analysis revealed that being employed reduced the risk of NEBF by 64% (OR: 0.36; IC: 0.28–0.40), as did good Spanish proficiency by 71% (OR: 0.29; IC: 0.20–0.51) and access to basic services by 68% (OR: 0.32; IC: 0.22–0.48) (Figure S1: Forest plot based on odds ratios of maternal factors associated with non-exclusive breastfeeding of Haitian immigrant women in southern Chile).

## 4. Discussion

There is currently a wealth of long-standing research that positions BF as an essential practice for achieving maternal, child, and population health; however, there are still difficulties in increasing BF rates. Therefore, understanding its prevalence and risk factors is one of the priorities in the study of BF [21].

The prevalence of EBF (54.3%) found in our study was higher than that reported in other immigrant groups. Vanderlinden et al. [22] observed that the prevalence of EBF varied between 25.5% and 50.0% among immigrants in Belgium; this was similar to findings by Tavoulari et al. [23] and Dennis et al. [24] in studies of immigrants in Greece (24.5%) and Chinese women in Canada (26.8%), respectively. A higher prevalence of EBF has been reported for women in Latin America and the Caribbean (59.7%) compared with other regions; Asian women have shown a lower prevalence of EBF and higher levels of pre-lacteal feeding [25,26].

Haitian women had a prevalence of EBF similar to that found in the host country than in their country of origin [10,11]. Likewise, our findings showed that the EBF rate increased as the duration of residency increased, while women with <12 months residency increased the risk of NEBF (OR: 2.2; CI: 2.09–2.78); this is similar to values reported by Wan et al. [27]. Nolan and Layte [11] studied immigrants in Ireland and found that BF rates tended to converge toward the rates of the host country and were directly proportional to the duration of residency. While Haitian immigrant women in Chile increased their prevalence of BF to the level of the host country, Gibson–Davis and Brooks–Gunn [28] showed that for each year of residency of immigrant women in the United States, the prevalence of BF decreased by 4%, which was closer to the rates of women born in the United States. This concurs with studies by Pak–Gorstein et al. [29] of African immigrant women in the United States.

We found higher rates of EBF in women with legal immigration status and employment, while those who were undocumented had up to 2.2 times more risk of NEBF. Hunter–Adams et al. [30] published similar results in a study of undocumented, unemployed immigrants in South Africa with low EBF rates. However, this differs from Brenne et al. [31], who found no relationship between EBF and immigrant status. The legal guarantees granted by the host country for access to public services and economic aid to immigrant groups influence EBF practices. In Chile, legal residency provides a person with access to remunerated employment, which is covered by Chilean legislation (Law 20.545) and allows a full 24 wk of postnatal leave. Abubakar et al. [32] in their study with immigrant women in Portugal indicated that formal paid jobs among immigrants promoted EBF.

Maternal education is a variable that in itself has a strong and positive association with BF [33]. In the present study, women with >12 years educational attainment (77.5%) and intermediate-high knowledge of BF (72.8%) had higher EBF rates. These results concur with findings reported by Wan et al. [27], Ogbo et al. [34], and Xiao et al. [35] in national and international studies of immigrants. We also found that low educational attainment (OR: 1.78; IC: 1.61–2.11) and poor knowledge of BF (OR: 1.96; IC: 1.81–2.27) increased the risk of NEBF almost twofold in Haitian immigrant women. Celi et al. [36] and Pitikultang et al. [37] concluded that immigrant women with higher educational attainment (OR: 6.1; IC: 3.6–10.3) and better knowledge of BF (OR: 2.2; IC: 1.1–4.4) increased the probability of EBF. 

Our results reveal that access to basic services, good language proficiency, and employment decreased the risk of NEBF up to 70%. Knowledge of the host country’s language is an important determinant of health. This has been described as one of the main barriers to BF practice and health care [38]. Lisi et al. [39] reported that immigrants in Portugal who were fluent in the language initiated EBF more frequently than other groups. As for access to basic services, our results concur with those shown by Pitikultang et al. [37], who concluded that access to basic services decreased by 40% the probability of NEBF among immigrants in Thailand.

## 5. Conclusions

In conclusion, Haitian immigrant women without legal residency, recently arrived, with low educational attainment, and poor knowledge of breastfeeding have between 1.78 and 2.34 times more risk of not providing exclusive breastfeeding to their children during the first 6 mo of life than those who do not exhibit these factors. However, access to basic services, an adequate knowledge of Spanish, and being employed are protective factors for exclusive breastfeeding (OR: 0.29–0.36). We highlight that this is the first study that has examined exclusive breastfeeding in Haitian immigrant women living in southern Chile. 

## Figures and Tables

**Table 1:** Sociodemographic characterization of the study sample.

Variable	*n* = 173
Age	
<35 years old	118 (68.2)
≥35 years old	55 (31.8)
Marital status	
Married or common-law partner	37 (21.4)
Single	136 (78.6)
Educational attainment	
≤12 years	39 (22.5)
>12 years	134 (77.5)
Parity (number of live births)	
1	75 (43.4)
2	58 (33.5)
≥3	40 (23.1)
Residency in Chile	
≤12 months	32 (18.5)
>12 months	141(81.5)
Immigration status	
Undocumented	36 (20.8)
Permanent residency	137 (79.2)
Employed	
Yes	130 (75.1)
No	43 (24.9)
Limited Spanish proficiency	
Yes	114 (65.9)
No	59 (34.1)
Access to basic services	
Yes	136 (78.6)
No	37 (21.4)
Weight status	
Normal	55 (31.8)
Overnutrition	118 (68.2)
Knowledge of breastfeeding	
Score < 8	47 (27.2)
Score ≥ 8	126 (72.8)
Exclusive breastfeeding	
<6 months	79 (45.7)
≥6 months	94 (54.3)

Data expressed as frequency (%).

**Table 2:** Relationship between maternal factors and breastfeeding of Haitian immigrant women in southern Chile.

Variable	NEBF (*n* = 79)	EBF (*n* = 94)	*p*
Age			
<35 years old	51 (64.6)	67 (71.3)	0.413
≥35 years old	28 (35.4)	27 (28.7)	
Marital status			
Married or common-law partner	14 (17.7)	23 (24.5)	0.353
Single	65 (82.3)	71 (75.5)	
Educational attainment			
≤12 years	24 (30.4)	15 (15.9)	0.029
>12 years	55 (69.6)	79 (84.1)	
Parity (number of live births)			
1	33 (41.8)	42 (44.7)	0.250
2	28 (35.4)	30 (31.9)	
≥3	18 (22.8)	22 (23.4)	
Residency in Chile			
≤12 months	21 (26.6)	11 (11.7)	0.018
>12 months	58 (73.4)	83 (88.3)	
Immigration status			
Undocumented	23 (29.1)	13 (13.8)	0.008
Permanent residency	56 (70.9)	81 (86.2)	
Employed			
Yes	53 (67.1)	77 (81.9)	0.034
No	26 (32.9)	17 (18.1)	
Limited Spanish proficiency			
Yes	45 (57.0)	69 (73.4)	0.025
No	34 (43.0)	25 (26.6)	
Access to basic services			
Yes	57 (72.2)	79 (84.0)	0.065
No	22 (27.8)	15 (16.0)	
Nutritional status			
Normal	24 (30.4)	31 (33.0)	0.745
Overnutrition	55 (69.6)	63 (67.0)	
Knowledge of breastfeeding			
Score < 8	28 (35.4)	19 (20.2)	0.027
Score ≥ 8	51 (64.6)	75 (79.8)	

NEBF: Non-exclusive breastfeeding; EBF: exclusive breastfeeding. Data expressed as frequency (%). χ^2^ test, significance *p* < 0.05.

**Table 3:** Logistic regression model for the association between maternal factors and non-exclusive breastfeeding of Haitian immigrant women in southern Chile.

Independent Variable	OR Raw (95% CI)	OR Adjusted (95% CI)
Risk factors		
Immigration status (undocumented)	2.56 (1.21–5.52)	2.34 (2.18–2.83)
Residency in Chile (<12 months)	2.76 (1.22–6.13)	2.23 (2.09–2.78)
Knowledge of breastfeeding (score < 8)	2.17 (1.11–4.33)	1.96 (1.81–2.27)
Educational attainment (≤12 years)	2.29 (1.15–4.84)	1.78 (1.61–2.11)
Protective factors		
Have employment	0.45 (0.22–0.91)	0.36 (0.28–0.40)
Have access to basic services	0.49 (0.23–1.03)	0.32 (0.22–0.48)
Spanish proficiency	0.48 (0.25–0.91)	0.29 (0.20–0.51)

Adjusted for age.

## Data Availability

The data presented in this study are available on request from the corresponding author.

## Supplementary Materials

The following supporting information can be downloaded at: https://www.mdpi.com/article/10.3390/nu14153173/s1, Figure S1: Forest plot based on odds ratios of maternal factors associated with non-exclusive breastfeeding of Haitian immigrant women in southern Chile.
