# Supplementary material for: Maternal Factors Associated with Non-Exclusive Breastfeeding in Haitian Immigrant Women in Southern Chile
Source: Nutrients. 2022 Aug 2;14(15):3173. doi: 10.3390/nu14153173 (PMC9370358; doi:10.3390/nu14153173)

Figure S1. Forest plot based on odds ratios of maternal factors associated with non-exclusive breastfeeding of Haitian immigrant women in southern Chile.

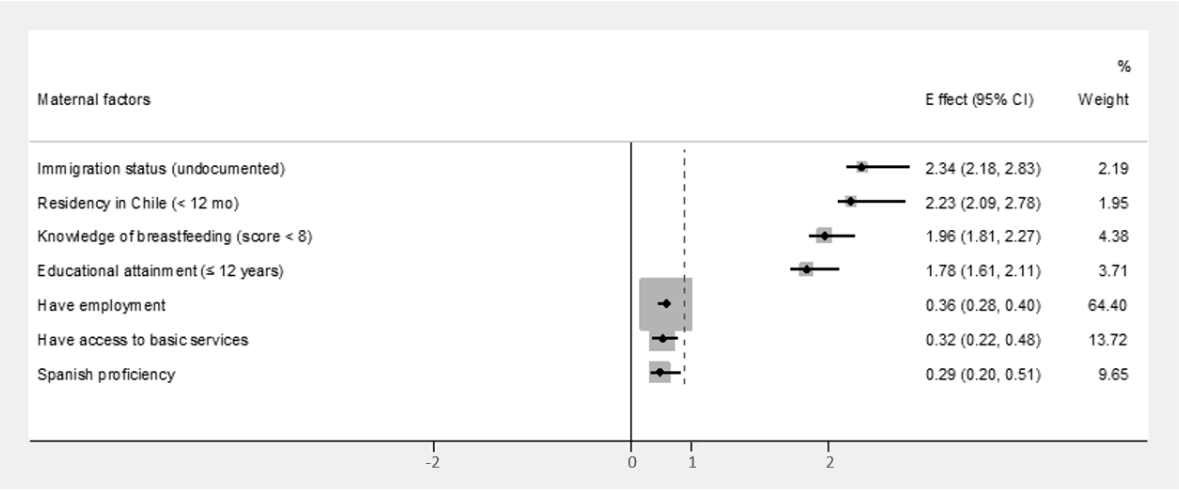

Supplement: Supplementary file 1 [file nutrients-14-03173-s001.zip › nutrients-1825957-supplementary.pdf]
